# Deep Learning Driven Noise Reduction for Reduced Flux Computed Tomography

**DOI:** 10.3390/s21051921

**Published:** 2021-03-09

**Authors:** Khalid L. Alsamadony, Ertugrul U. Yildirim, Guenther Glatz, Umair Bin Waheed, Sherif M. Hanafy

**Affiliations:** 1 College of Petroleum Engineering and Geosciences (CPG), King Fahd University of Petroleum and Minerals (KFUPM), Dhahran 31261, Saudi Arabia; g201265840@kfupm.edu.sa (K.L.A.); umair.waheed@kfupm.edu.sa (U.B.W.); sherif.mahmoud@kfupm.edu.sa (S.M.H.); 2 Institute of Applied Mathematics, Middle East Technical University (METU), Ankara 06590, Turkey; ertugrul.yildirim@metu.edu.tr

**Keywords:** deep learning, convolutional neural network (CNN), computed tomography (CT) images, micro-CT, reduced exposure time, efficient CT measurement, enhanced temporal resolution, improved image quality, rock (porous medium) images, ASTRA toolbox

## Abstract

Deep neural networks have received considerable attention in clinical imaging, particularly with respect to the reduction of radiation risk. Lowering the radiation dose by reducing the photon flux inevitably results in the degradation of the scanned image quality. Thus, researchers have sought to exploit deep convolutional neural networks (DCNNs) to map low-quality, low-dose images to higher-dose, higher-quality images, thereby minimizing the associated radiation hazard. Conversely, computed tomography (CT) measurements of geomaterials are not limited by the radiation dose. In contrast to the human body, however, geomaterials may be comprised of high-density constituents causing increased attenuation of the X-rays. Consequently, higher-dose images are required to obtain an acceptable scan quality. The problem of prolonged acquisition times is particularly severe for micro-CT based scanning technologies. Depending on the sample size and exposure time settings, a single scan may require several hours to complete. This is of particular concern if phenomena with an exponential temperature dependency are to be elucidated. A process may happen too fast to be adequately captured by CT scanning. To address the aforementioned issues, we apply DCNNs to improve the quality of rock CT images and reduce exposure times by more than 60%, simultaneously. We highlight current results based on micro-CT derived datasets and apply transfer learning to improve DCNN results without increasing training time. The approach is applicable to any computed tomography technology. Furthermore, we contrast the performance of the DCNN trained by minimizing different loss functions such as mean squared error and structural similarity index.

## 1. Introduction

Computed tomography has been recognized as an indispensable technology not only in the healthcare domain but also with respect to industrial applications such as reverse engineering [1,2], flaw detection [3] and meteorology to name a few [4,5]. The non-destructive nature of CT scanning has also proven to be tremendously valuable in the case of geomaterials, allowing to elucidate transport phenomena in porous media; visualize deformation and strain localization in soils, rocks or sediments; or perform fracture and damage assessment in asphalt, cement and concrete [6]. The three-dimensional data obtained helps to better inform numerical models improving their predictive power and enables delineation of physical properties of the specimen under investigation. Both qualities are particularly valued in the area of digital rock physics [7,8].

The technology has, however, shortcomings, in particular with respect to monitoring dynamic processes. The limitation of prolonged acquisition times is distinctively more severe in the case of micro-CT technologies, where it may take several hours for a scan to complete. During acquisition, the object should not physically change—or change as little as possible—to allow for meaningful reconstruction of the sinograms. Medical CT scanners, per design, offer significantly shorter acquisition times, mere minutes depending on the sample size. Certain experiments, however, stand to benefit greatly from increased exposure times as noise is decreased. The noise reduction gives rise to better statistics if, for example, porosity is to be estimated in combination with a non-wetting radio contrast agent [9]. Similarly, core flood experiments necessitate the presence of a vessel to maintain temperatures and pressures resulting in attenuation of the X-rays. Again, prolonged acquisition times, combined with high tube voltages and currents, yield a better image quality. From experience, scanning of a one-inch long rock specimen using a medical CT scanner operating at maximum tube voltage (140–170 kV), current (200 mA) and exposure time (4 s per slice) requires up to 30 min between scans to allow for the X-ray tube to cool down. Conversely, certain reactive processes happen rather rapidly, mandating low exposure times if the dynamics are to be captured. This is particularly true for high-temperature experiments given the exponential dependency of the reaction rate on heat [10,11].

The medical domain of low dose computed tomography (LDCT) seeks to reduce the exposure time in an effort to minimize the radiation risk [12]. Lowering the flux by reducing the exposure time, tube peak voltages and currents will inevitably decrease the image quality and, thereby, the diagnostic value [13]. Photon emission from the X-ray source is modeled as a Poisson process [14] and photon starvation at the detector gives rise to Poisson noise [15,16]. Additional noise is introduced during the quantization of the signal and in the form of electronic noise [17]. Naturally, researchers have sought to reduce artifacts employing improved algorithms during the reconstruction of the 3D data from the projections/sinograms [18] or post-reconstruction. Generally, the latter approach is more common given that the raw CT data are often not accessible, especially in the case of a medical CT system [19,20].

Conventional signal processing techniques require a good understanding of the underlying nature of noise to optimize the filter design. Noise statistics, to guide the filter model, may be collected experimentally, but, from experience, this constitutes a rather laborious process.

The application of deep neural networks in combination with computed tomography of geomaterials is rapidly gaining momentum. Reviewing the literature, researchers are particularly interested in applying super-resolution concepts or exploiting DCNNs for segmentation and rock classification purposes [21,22,23,24,25]. Conversely, our primary focus lies on the reduction of scan time while maintaining, if not improving, image quality.

Deep convolutional neural networks (DCNNs) have been successfully applied in the medical domain to map low-quality, low-dose images to higher-dose, higher-quality images [26,27]. In this paper, we seek to build on this general approach and apply it to computed tomography scanning of geomaterials for numerous reasons. First, the reduction of acquisition time allows for an increase in the temporal resolution. Consequently, experiments previously deemed out of reach due to the associated dynamics can now be entertained. This is of main interest to the authors, given that we specialize in the design of high-pressure/high-temperature core holders for reactive processes for both medical and micro-CT technologies. To reiterate, reaction kinetics are governed by an exponential temperature dependency. Thus, it is of utmost importance to reduce scan time to capture the dynamics. Second, with respect to digital rock physics, the accuracy of the estimated rock properties strongly depends on the image quality [28,29,30]. Third, high quality images are a prerequisite for resolution enhancement techniques [31,32,33]. In addition, a reduction in scan time will contribute to an increase in the lifetime of the X-ray tube (medical CT) or the filament (micro-CT).

Using artificial rock CT images derived from simulated parallel-beam projections, Pelt et al. [34] obtained promising results with respect to improved image quality by means of DCNNs [34]. The work presented in this paper seeks to extend the DCNNs filtering concept including results not only for synthetic data created using the ASTRA toolbox [35] but also for actual micro-CT data generated by a *FEI Heliscan microCT* operating with a cone-beam. Importantly, we not only aim to reduce scanning time but also seek to improve the quality of the reconstructed images compared to the high dose training images, simultaneously.

In short, in this paper, we study two deep learning architectures for improving degraded rock images resulting from reduced exposure time micro-CT scans. Furthermore, we investigate the applicability of transfer learning to minimize the number of training images needed. In addition, we explore the impact of mean-squared error (MSE) and structural similarity index measure (SSIM) loss functions on the reconstructed image quality. While both loss functions are capable of considerably improving the respective quality metrics, PSNR and SSIM, they tend to emphasize different structural features. These findings are crucial for improving digital rock physics applications where rock properties, such as porosity and permeability, are to be estimated from computed tomography data only.

## 2. Methods

Convolutional neural networks (CNNs) constitute a subset of artificial neural networks (ANNs), heavily relying on digital filter operations (kernel/convolution matrix), where the weights of the filters are informed during the training process to minimize a particular loss metric between the predictions and the training (true) samples. CNNs are especially suitable for computer vision applications given that the filters can capture the spatial relation between individual pixels or image elements.

The main components of CNNs are convolution layers and activation functions. Convolution layers consist of filters that slide across the input feature map (e.g., image). Each element of a filter is multiplied by the overlapping element of the input feature map and subsequently summed to yield one element of the output feature map. This operation is followed by activation functions to add non-linearity, empowering a CNN to learn the complex relationship between inputs and their corresponding labels. A common activation function is the rectified linear unit (ReLU), designed to set negative values to zero and linearly map inputs to outputs in case of positive values.

### 2.1. Details of Network Architectures Investigated

**Figure 1:**
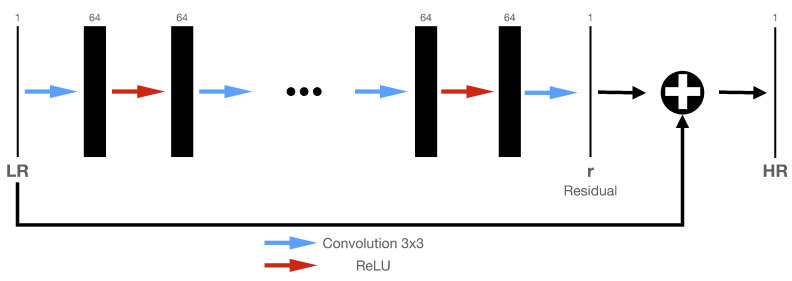
VDSR architecture showing cascaded pair of layers. The input is a low-resolution image, or a noisy image in our case, which goes through layers and gets transformed to a high-resolution or denoised image. The convolutional layers use 64 filters each.

For the transfer learning aspect of this work, we take advantage of the Very Deep Super Resolution (VDSR) architecture and the associated pretrained VDSR by Kim et al. [36]. Both were obtained from the MathWorks website [37]. The VDSR architecture consists of 20 weighted convolution layers followed by a ReLU. Each convolution layer, except the final and input layers, accommodates 64 filters of size 3 × 3. Figure 1 shows the network architecture.

**Figure 2:**
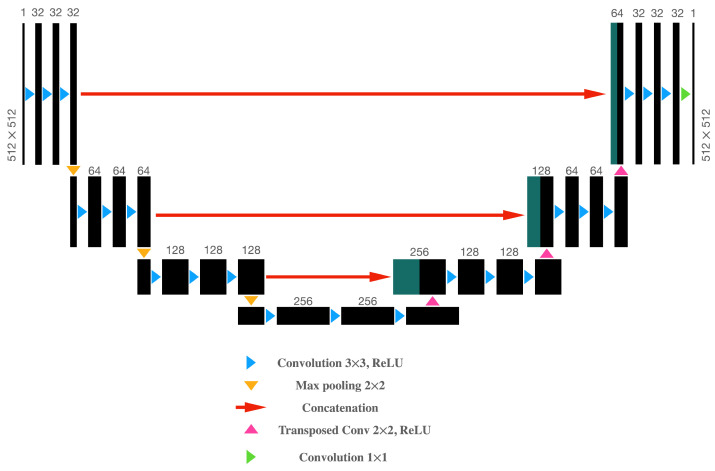
Architecture of the proposed DCNN (U-Net), which is based on a residual encoder–decoder structure. Each black box represents a feature map. The number of channels is denoted at the top of each box. The input and output images have the same size (height and width), which is indicated at the sides of the first and last box. Dark green boxes represent copied feature maps from the encoder block. The arrows state different operations.

The second architecture investigated constitutes a deep convolutional neural network (DCNN) based on a residual encoder–decoder architecture, known as the U-Net network. The encoder or feature extractor component consists of three blocks, with each block comprising three consecutive convolution layers where all layers are followed by a ReLU activation function. At the end of each block, a sample-based discretization process in the form of a max pooling operation is executed to reduce the size of the feature map. Similarly, the decoder incorporates a transposed convolution layer followed by three consecutive convolution layers to be terminated by ReLU activation functions. In addition, skip connections between each encoder block and its corresponding decoder block are included to concatenate the output of the transposed convolution layers with the feature map from each encoder block. All convolution layers, except the last layer, are of size 3 × 3. In the encoder part, the number of filters increases for each block (32, 64 and 128, sequentially). Equivalently, in the decoder part, the number of filters decreases for each block (128, 64, and 32, sequentially). The network configuration is outlined in Figure 2.

### 2.2. Loss Functions

The MSE is a commonly used loss function for image restoration tasks and is defined as follows:

(1) $$L^{\mathrm{MSE}}\left(P\right)=\frac{1}{N}{\sum_{p\in P}\left[t\left(p\right)-r\left(p\right)\right]}^{2},$$

where *p* constitutes the index of the pixel in patch *P*, t(p) is the pixel value in the trained patch, r(p) corresponds to the pixel value of the reference image and *N* is the number of pixels in a given patch [38].

The structural similarity index measure (SSIM), however, is often regarded as a more pragmatic metric for evaluating image quality, particularly with respect to human visual perception [39]. For a pixel *p*, the SSIM is given as follows:

(2) $$\mathrm{SSIM}\left(p\right)=\frac{\left(2\mu_{r}\mu_{t}+c_{1}\right)\left(2\sigma_{tr}+c_{2}\right)}{\left(\mu_{r}^{2}+\mu_{t}^{2}+c_{1}\right)\left(\sigma_{r}^{2}+\sigma_{t}^{2}+c_{2}\right)},$$

where μ and $\sigma^{2}$ reflect the average and variance of the training and reference patch, respectively, and $\sigma_{tr}$ is the associated covariance. $c_{1}$ and $c_{2}$ are constants required to avoid division with a weak denominator and partially depend on the dynamic range of the pixels. Given that the SSIM range is bounded, SSIM ≤ 1, with 1 being indicative that the training image is identical to the reference image, the loss function needs to be written as follows:

(3) $$L^{\mathrm{SSIM}}\left(P\right)=\frac{1}{N}\sum_{p\in P}1-\mathrm{SSIM}\left(p\right).$$

For training of the VDSR and the pretrained VDSR, an image patch size of 41 × 41 with 128 patches per image was utilized. The Adam optimizer was configured for a learning rate of 0.0001, 5 epochs and a mini-batch size of 32. For this case we only applied the MSE loss function as defined in Equation (1).

Both loss functions were exploited to train the DCNN (U-Net), employing an image patch size of 512 × 512, 1600 training images, 400 testing images, the Adam optimizer configured for a learning rate of 0.0001, 50 epochs and a mini-batch size of 8.

Naturally, for both architectures, the low-quality/low-exposure images served as input to be trained on the corresponding high-quality/high-exposure scans.

### 2.3. Data Acquisition Details

Using a *FEI Heliscan microCT*, configured to perform 1800 projections per revolution at a tube voltage of 85 kV and a current of 72 mA, two datasets were acquired at an exposure time of 0.5 and 1.4 a, respectively. Consequently, for 1800 projections per revolution, the reduction in exposure time reduces acquisition time per revolution from 42 to 15 min, a decrease of more than 60%.

As mentioned above, an increased exposure time translates to a greater image quality given that more photons are collected at the detector, thereby decreasing the noise. Henceforth, we refer to the scans collected at 1.4 s as high quality images and 0.5 s data as low quality images. The scanned specimen was a carbonate of unknown origin measuring 1.5 inches in diameter and about 2 inches in length. The sample geometry dictated a minimum voxel size of about 14 μm. The sample was chosen because the pore space is sufficiently large to be adequately resolved by the micro-CT.

**Figure 3:**
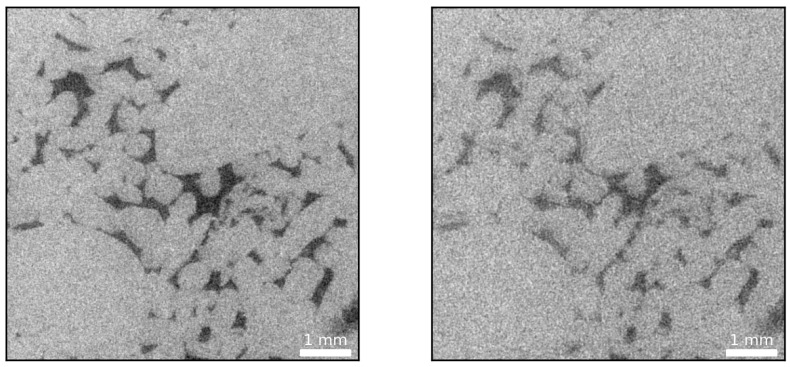
High exposure time, 1.4 s, CT image (**left**) and low exposure time, 0.5 s, CT image (**right**) of a carbonate rock sample where dark colors are indicative of pore space. Evidently, a reduced exposure time results in an increased noise level owing to the photon starvation at the detector.

During all scans, an approximately 100 μm thick aluminum sheet was mounted at the tungsten target window to soften the X-rays in an effort to minimize beam hardening artifacts. The amorphous-silicon, large-area, digital flat-panel detector with 3072 × 3072 pixels, is capable of supporting a pixel array of nine mega-pixels with a dynamic range of 16 bits. The effective scan resolution was 2884 × 2884 pixels. To accommodate the network architecture, the individual slices needed to be split into tiles of size 512 × 512 pixels and the gray-scale values were normalized to a range between zero and one. In a first-order approximation, the gray values may be interpreted as density values where brighter areas indicate larger density values and darker areas suggest lower density values. Hence, pore space is represented by shades of black (see, e.g., Figure 3).

### 2.4. ASTRA Toolbox

As shown below, the images predicted by the network are of significantly greater quality than the training images collected at an exposure time of 1.4 s. Consequently, it became necessary to create an artificial case based on the images predicted by the network to verify that the architecture is indeed predicting the ground truth.

The ASTRA toolbox is an open-source software for tomographic projections and reconstruction, available for MATLAB© and Python [35,40]. Throughout this study, MATLAB© 2020a in combination with the ASTRA toolbox V1.9 was utilized. The cone beam projection module in the ASTRA toolbox offers the following three reconstruction algorithms: FDK by Feldkamp et al. [41], simultaneous iterative reconstruction technique (SIRT) by Gilbert [42] and conjugate gradient least squares (CGLS) by Frommer and Maass [43].

The toolbox allowed us to simulate artificial low and high exposure time images based on images predicted by the VDSR network utilizing a cone beam geometry with 1800 projections per revolution. Details explaining the toolbox are provided elsewhere [44,45,46,47].

**Figure 4:**
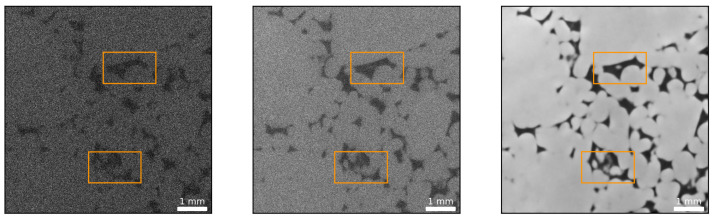
The image on the left is an example of a low exposure time slice with the image in the center being the high exposure time equivalent. The image on the far right is the reconstruction based on the SSIM optimized DCNN (U-Net). The DCNN performs remarkably well in reconstructing fine scale features, barely visible even in the case of a longer exposure time.

Given that the toolbox does not model noise sources and, effectively, assumes a perfect detector, Poisson noise mimicking the signal-to-noise ratio (SNR) values of the CT images was added to the projections to simulate the physical process at the detector. Subsequently, the sinograms were reconstructed by means of the FDK algorithm to yield artificial low and high exposure time scans. To summarize, at this point, the following image series are available:

1. A 0.5 s exposure time series obtained from the micro-CT, constituting the low quality data (see, e.g., right-hand-side in Figure 3)
2. A 1.4 s exposure time series obtained from the micro-CT, representing the high quality data (see e.g., left-hand-side in Figure 3)
3. The images predicted by the network that are of greater quality (see, e.g., Figure 4) compared to the 1.4 s exposure training images (labels) (these images subsequently serve as the ground truth from which artificial low and high exposure time series were obtained)
4. An artificial low quality image series derived from predicted images using the ASTRA toolbox mimicking the results obtained from the micro-CT in terms of SNR at an exposure time of 0.5 s
5. An artificial higher quality image series, delineated from predicted images using the ASTRA toolbox resembling the results obtained from the micro-CT in terms of SNR at an exposure time of 1.4 s

The artificially created series was used to validate the predictive power of the trained network, as detailed in Section 3.4.

## 3. Results and Discussion

In this section, we benchmark the proposed DCNNs to restore low quality micro-CT images as a result of reduced exposure times. We begin by highlighting the problem and its adverse consequences on the scanned image quality. Next, we explore the applicability of transfer learning to help expedite the training of the PVDSR network.

Exploiting a pre-trained VDSR network, we substantiate that optimal performance can be obtained more quickly than relying on a randomly initialized VDSR network. In addition, we also compare the reconstruction performance of different loss functions, including MSE and SSIM. Finally, we prove the predictive power of the DCNNs by testing them against simulated low- and high-exposure images from the ASTRA toolbox [35].

### 3.1. Reduced Exposure

In the context of rock imaging or imaging of materials in general, the reduction of exposure time offers three main advantages.

Firstly, micro-CT scanning, if offered as a commercial service, from experience, is charged on an hourly basis ranging from hundreds to thousands of dollars per hour. Evidently, high quality scans necessitate a longer exposure time, consequently being more costly. Thus, a decrease in scan time while maintaining image quality is beneficial to both parties: it allows the provider to offer the service to the client at a reduced cost and, at the same time, increase the throughput.

Secondly, any reduction in exposure time will result in a more economic use of the filament life time. Generally, a single filament costs about 700 to 1000 dollars and is rated for about 300 working hours. Assuming the particular scan time reduction achieved in this work, roughly 60%, the filament lifetime may be increased up to 480 working hours. In addition, as shown below, the network also performs exceedingly well at denoising the image without the need for user intervention.

Thirdly, and most importantly, a reduction in exposure time renders the technology available to elucidate processes previously out of reach due to the associated dynamics.

As mentioned above, a reduction in exposure time increases the noise level due to photon starvation at the detector. For example, Figure 3 illustrates how reduced exposure time CT data (0.5 s) yields a considerably noisier image compared to a 1.4 s exposure time scan.

The noise present in the low exposure, and, noticeably, also in the high-exposure image, is problematic if rock properties such as porosity and permeability are to be estimated. Accurate porosity values strongly rely on the ability to distinguish between the solid phase and the pore space, precisely. With respect to Figure 3, this can constitute a daunting task, especially for the low exposure time case. Commonly, a median or smoothing filter followed by, for example, a histogram or watershed based segmentation is applied [48].

Estimation of permeability is significantly more involved as it necessitates a computational fluid dynamics study on the segmented data [49]. In addition, permeability is particularly dependent on fine scale features and mineralogy in the case of wetting fluids.

To address the aforementioned challenges, we sought to train DCNNs to denoise low-exposure micro-CT images without the need for expert knowledge with respect to filter design.

### 3.2. Transfer Learning

While DCNNs have shown remarkable performance for myriad scientific problems, they are well-known for being data- and resource-intensive due to a large number of trainable parameters. Lack of training samples or computational resources may hurt the performance of these networks in either of these situations. A pragmatic approach to address this issue is to take advantage of transfer learning, a machine learning technique seeking to apply previously gained knowledge to speed up finding the solution to a different yet related problem.

In this particular study, we explore the applicability of transfer learning using the VDSR network, as illustrated in Figure 1, and compare the reconstruction performance of the VDSR network initialized as per [50] with a pre-trained VDSR network by minimizing the MSE as defined in Equation (1). We train both the pre-trained VDSR network and the VDSR network for a range of the number of training images, starting with 50 training images up to a maximum of 300 training images. For each particular number of training images, we measure the reconstruction performance of the two networks by comparing the average SSIM and peak signal-to-noise ratio (PSNR) values for the pre-trained and for the randomly initialized network) derived from 400 test images.

**Figure 5:**
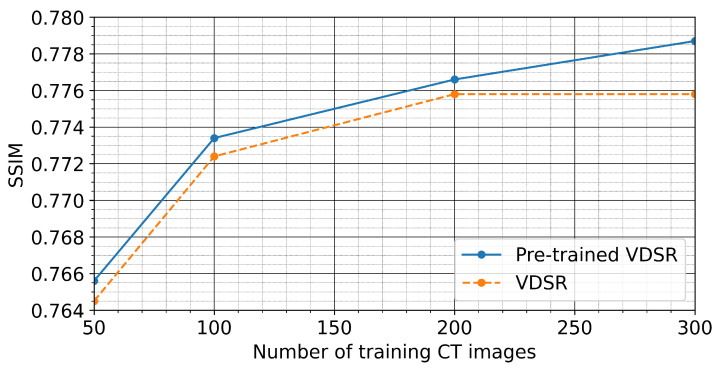
Summary plot of the average SSIM values employing 400 test images as predicted by the pre-trained VDSR network and a VDSR initialized following the approach of He et al. [50]. From 50 to 200 training images, both networks show similar performance gains with respect to the corresponding SSIM values. After 200 training images, however, the pre-trained network further increases the SSIM value compared to the VDSR. In general, however, the pre-trained VDSR yields greater SSIM values for all cases.

**Figure 6:**
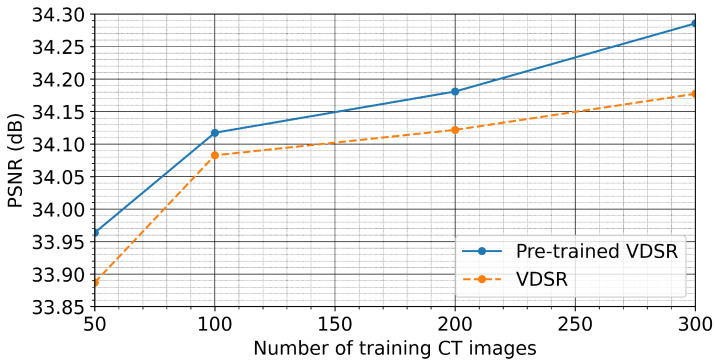
Summary plot of the average PSNR values employing 400 test images as predicted by the pre-trained VDSR network and the VDSR. From 50 to 100 training images, the VDSR shows a slightly better improvement compared to the pre-trained VDSR network. This advantage diminishes as the number of training images increases. Similar to the SSIM plot shown in Figure 5, the pre-trained VDSR yields greater SSIM values for all cases.

**Figure 7:**
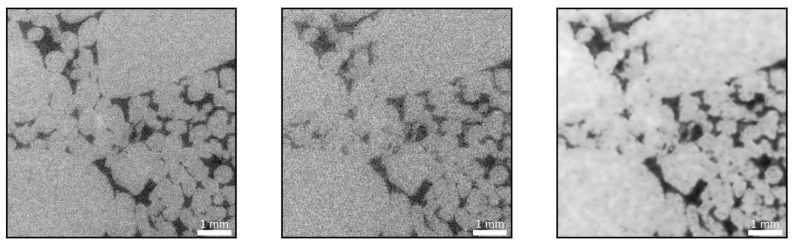
From left to right: High exposure (reference) image, low-exposure image (SSIM = 0.54, PSNR = 23 dB) and denoised image (SSIM = 0.78, PSNR = 34 dB) using the pre-trained VDSR network.

Figure 5 and Figure 6 show that the pre-trained VDSR network always yields a better overall reconstruction performance for a given number of training images. This holds true for both considered metrics to quantify reconstruction quality (SSIM and PSNR), demonstrating the inherent advantage of transfer learning. Figure 7 exemplifies the reconstruction quality achieved by the pre-trained VDSR network, based on 300 training images, improving average SSIM and PSNR values of the low-exposure image from 0.54±0.02 and 23±0.57 dB to 0.78±0.02 and 34±0.57 dB, respectively.

Examining the predicted image in Figure 7, it should be noted that it is indeed of greater quality (less noisy) than the high quality reference image or training label. The noise is greatly reduced and the grain boundaries show a sharper delineation. This is somewhat surprising given that, from a conventional signal processing point of view, both edges and noise constitute high frequency content. Often, filters designed to remove high frequency content are often found to smear out edges and subtle details [51]. Granted, median filters, or filters utilizing local statistics, in general, perform well in preserving them, yet it is remarkable that the network learned to differentiate between discontinuities in the form of edges and noise. This particular aspect is addressed in more detail in Section 3.4.

### 3.3. Loss Functions

Proper selection of the loss, objective or fitness function is crucial in guiding the learning process of the network. The MSE loss function, as defined in Equation (1), is a preferred metric to optimize the weights owing to its simplicity and well-behavedness with respect to gradient calculations. Notably, the minimization of the MSE indirectly maximizes the PSNR.

**Figure 8:**
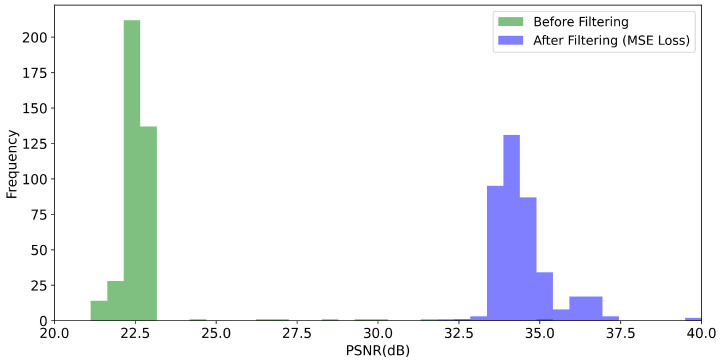
Histogram of the PSNR values obtained for the 400 test images. “Before filtering” refers to the low exposure scans. “After filtering” refers to the DCNN (U-Net) denoised scans where the network as optimized with respect to the MSE loss function.

**Figure 9:**
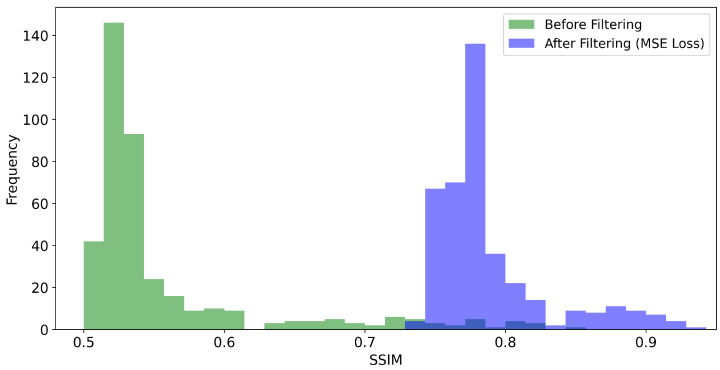
Histogram of the SSIM values obtained for the the 400 test scans. “Before filtering” refers to the low-exposure images. “After filtering” refers to the DCNN (U-Net) denoised scans where the network was optimized with respect to the MSE loss function.

**Figure 10:**
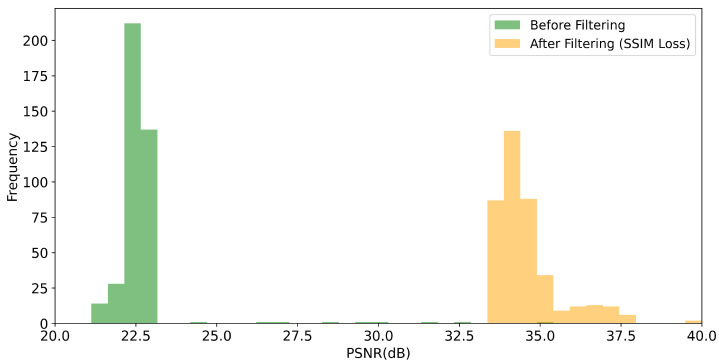
Histogram of the PSNR values obtained for the 400 test images. “Before filtering” refers to the low exposure scans. “After filtering” refers to the DCNN (U-Net) denoised scans where the network was optimized with respect to the SSIM loss function.

**Figure 11:**
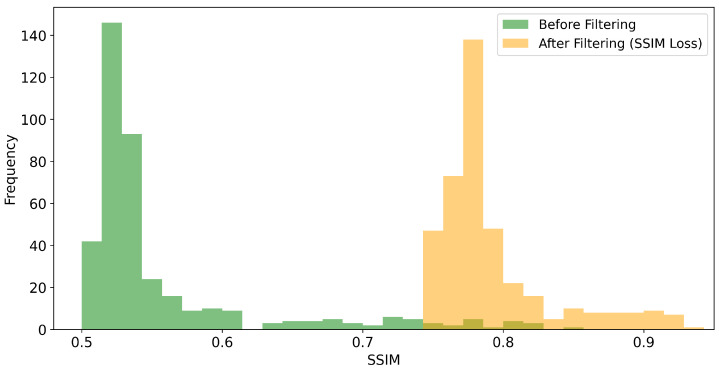
Histogram of the SSIM values obtained for the the 400 test scans. “Before filtering” refers to the low-exposure images. “After filtering” refers to the DCNN (U-Net) denoised scans where the network was optimized with respect to the SSIM loss function.

Given the particular problem of image prediction, we seek to compare the impact of the MSE loss function against the SSIM loss function, as defined in Equation (2), on the image quality metrics PSNR and SSIM, respectively. For this purpose, we trained the U-net-derived DCNN on 1600 training images for each metric. The trained networks were benchmarked using 400 test images. In general, we obtain considerable improvements for both the SSIM and PSNR values of the reconstructed images, as shown in Figure 8, Figure 9, Figure 10 and Figure 11. For the MSE optimized network, the PSNR increased, on average, from about 22.6 to 34.5 dB (see Figure 8) and the SSIM from 0.56 to 0.79 (see Figure 9). In the case of the SSIM optimized network, the PSNR increased, on average, to 34.6 dB (see Figure 10) and the SSIM to 0.79 (see Figure 11).

**Figure 12:**
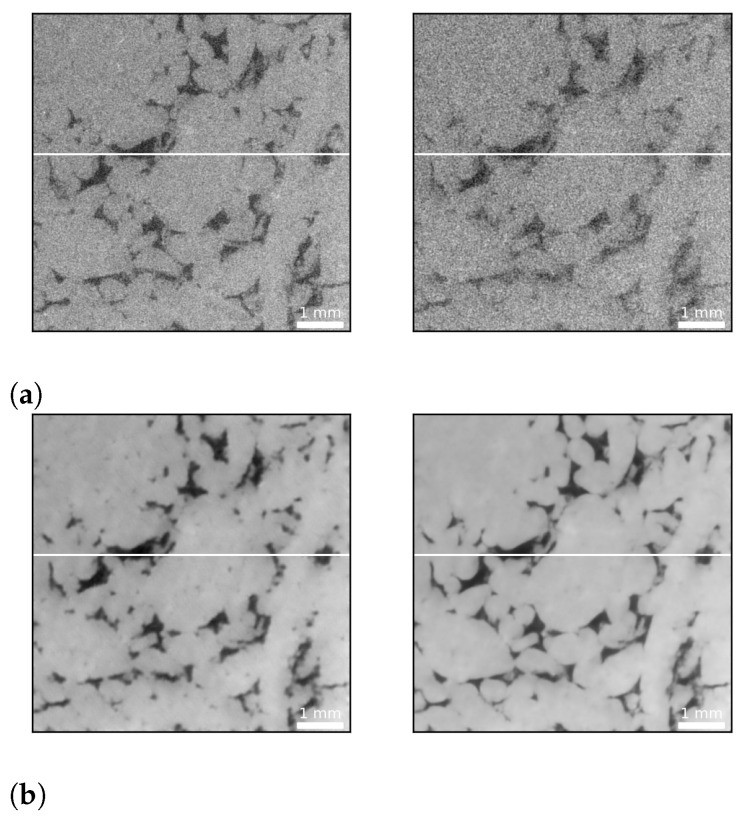
DCNN (U-Net) denoising example from the test set: (**a**) A pair of images from the test dataset. The image on the left is a high exposure time (1.4 s) scan, while the image on the right is the equivalent low exposure time (0.5 s) scan (SSIM = 0.52, PSNR = 22 dB). (**b**) Denoising results exemplifying the performance of the network. The left image shows the prediction of the DCNN optimized using the MSE loss function (SSIM = 0.77, PSNR = 34 dB), while the right image exhibits the prediction of the SSIM optimized network (SSIM = 0.77, PSNR = 34 dB). The MSE optimized network predicts coarser grain textures (greater variation in grayscale values indicative of larger variations in grain density) and boundaries and seems to be more sensitive to fine scale pore space (compare upper left quadrant of both images for the presence of fine scale pore space). Conversely, the SSIM optimized network suggests smoother textures, sharper grain boundaries and appears to be less sensitive to fine scale pore space. The white lines indicate the location of a horizontal profile to compare the network’s performance in the subsequent figure.

**Figure 13:**
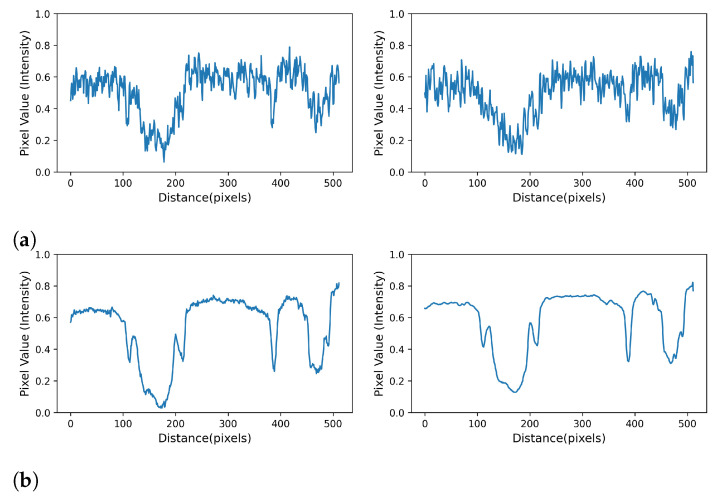
Intensity profiles taken along the white lines shown in Figure 12 that demonstrate the remarkable ability of the network to remove noise: (**a**) the profiles taken along the cross sections depicted in Figure 12a that represent the high- and low-exposure images respectively; and (**b**) the corresponding profiles that represent the MSE reconstructed (left) and SSIM reconstructed (right) images.

Both loss functions perform remarkably well in restoring fine scale features, as exemplified in Figure 4, and yield similar image quality improvements. With respect to Figure 12, however, it seems they tend to emphasize different features of the data. The MSE optimized network predicts coarser grain textures and boundaries and seems to be more sensitive to fine scale pore space. Conversely, the SSIM optimized network suggests smoother textures, sharper grain boundaries and appears to be less sensitive to fine scale pore space. Plotting the cross sections indicated in form of white lines in Figure 4, Figure 13 evidences the remarkable ability of the network to remove noise.

Surprisingly, the quality of the predicted image is clearly superior to the quality of the long exposure time image. As mentioned above, the network is seemingly able to distinguish between high frequency noise and discontinuities in the form of edges. At this point, it became necessary to verify the predictive power of the networks, and it was decided to create artificial cases where the ground truth is known. The approach is detailed in the next section.

### 3.4. ASTRA Toolbox

As elaborated in the previous section, the images predicted by the DCNNs (images on the right of Figure 7 and Figure 12b) are not only of superior quality compared to the low-exposure images but also exhibit less noise than their corresponding high-exposure images or the training labels. As discussed in the Introduction and substantiated by Figure 12b, noise can be reduced by increasing the exposure time or flux in general. In addition, the choice of the reconstruction algorithm is also critical. Iterative reconstruction algorithms such as Simultaneous Iterative Reconstructive Technique (SIRT) or Conjugate Gradient Least Squares (CGLS) are well known to suppress noise compared to classic filtered backprojection (FDP) via Feldkamp-type (FDK) reconstruction algorithms [52,53]. The particular algorithm employed by the *FEI Heliscan microCT* is proprietary.

**Figure 14:**
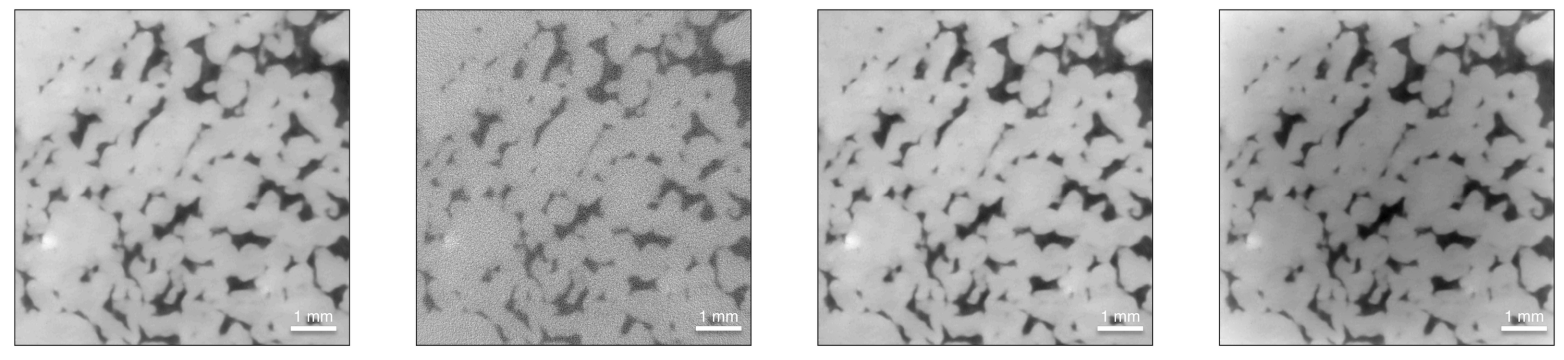
From left to right: Reference image (as predicted from the network representing the ground truth), FDK reconstruction, SIRT reconstruction and CGLS reconstruction.

Given the surprising results, we sought to verify them by creating an artificial dataset for which the ground truth is known. For this purpose, the VDSR network’s denoised images were fed into the ASTRA toolbox to create noisy projections mimicking low and high exposure time images. Next, the projections were reconstructed using FDK, SIRT and CGLS. As summarized in Figure 14, SIRT and CGLS performed well in removing the noise, whereas FDK failed to do so. Hence, we decided to solely focus on FDK for the creation of the artificial dataset. Subsequently, the artificial dataset was tested utilizing the trained networks.

**Figure 15:**
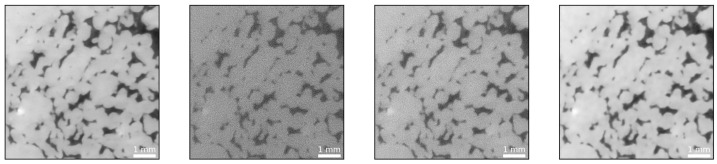
From left to right: Reference image (as predicted from the network representing the ground truth); artificial low-exposure image created via FDK, i.e., VDSR input (SSIM = 0.17, PSNR = 14 dB); artificial high-exposure image created, via FDK i.e., VDSR label (SSIM = 0.30, PSNR = 21 dB); and VDSR output (SSIM = 0.89, PSNR = 26 dB).

Figure 15 shows results for the VDSR network trained on the artificial datasets, i.e., it was trained to map the artificial low exposure to its corresponding artificial high exposure (training example/label). The average SSIM and PSNR values of the predicted images from the network (SSIM = 0.86, PSNR = 25 dB) are better than the artificial high- (SSIM = 0.28, PSNR = 18 dB) and low-exposure images (SSIM = 0.17, PSNR = 14 dB) according to 200 test images, where the reference images (ground truth) were used to calculate these values. It is, again, surprising that the output images of the network yield greater quality results compared to the training examples (artificial high-exposure images). This substantiates, however, the results reported in the previous section where the ground truth was unknown.

## 4. Conclusions

In this work, we successfully demonstrate the value of DCNN to improve the quality of micro-CT scans of a carbonate rock sample. The proposed method has the potential to reduce the exposure time without compromising the scan quality. Assuming 1800 projections per revolution, the acquisition time per revolution reduces from 42 to 15 min, a decrease of more than 60%. We found that the networks are able to predict images of superior quality compared to the long exposure time training images (labels). In particular, the networks are seemingly able to distinguish between unwanted high frequency content such as noise and actual high frequency features of the data such as discontinuities in the form of grain boundaries. Crucially, we verified the predictive power of the networks by creating a synthetic dataset, allowing us to compare against a known ground truth.

Given the substantial time requirements for training the networks, we also investigated the applicability of transfer learning. Using a pre-trained VDSR network, we found that high quality images can be obtained for a smaller number of training epochs compared to training from scratch.

Additionally, we highlighted the impact of MSE and SSIM based loss functions on the DCNN predictions. Both yield similar improvements with respect to PSNR and SSIM. They tend to, however, emphasize different structural aspects of the specimen. The MSE optimized network predicts coarser grain textures and boundaries and seems to be more sensitive to fine-scale pore space. Conversely, the SSIM optimized network suggests smoother textures, sharper grain boundaries and appears to be less sensitive to fine scale pore space.

To summarize, the proposed method enables substantial savings in acquisition time while simultaneously improving the scan quality. The reduction in scan time is an important aspect if dynamic processes are to be elucidated or higher sample throughput is required. Importantly, the approach is applicable to any computed tomography technology (medical CT, micro-CT and industrial CT). The vast improvement in image quality, without the need for expert intervention, is crucial for digital rock physics applications where rock properties such as porosity and permeability are estimated solely from computed tomography data. Inevitably, the accuracy of the estimation is dictated, in part, by the scan quality.

Nevertheless, we have to be cognizant of the limitations of the approach. The networks constitute, to a great extent, black boxes, making it challenging to implement improvements. In addition, we cannot discount the fact that a substantial amount of data is required to train the network even though transfer learning can help to reduce the volume required.
